# Effectiveness of exercise intervention and health promotion on cardiovascular risk factors in middle-aged men: a protocol of a randomized controlled trial

**DOI:** 10.1186/1471-2458-13-125

**Published:** 2013-02-11

**Authors:** Svetlana From, Helena Liira, Jenni Leppävuori, Taina Remes-Lyly, Heikki Tikkanen, Kaisu Pitkälä

**Affiliations:** 1 Helsinki University Central Hospital, Unit of Primary Health Care and University of Helsinki, Department of General Practice and Primary Health Care, Helsinki, Finland; 2 Department of Sports and Exercise Medicine, University of Helsinki and Foundation of Sports and Exercise Medicine, Helsinki, Finland; 3 Kirkkonummi Health Center, Kirkkonummi, Finland

**Keywords:** Exercise intervention, Health promotion, Metabolic syndrome, Exercise activity

## Abstract

**Background:**

Although cardiovascular disease has decreased, there is still potential for prevention as obesity and diabetes increase. Exercise has a positive effect on many cardiovascular risk factors, and it can significantly reduce the components of metabolic syndrome. The main challenge with exercise in primary care is how to succeed in motivating the patients at risk to change and increase their exercise habits. The objective of this study is to modify the cardiovascular risk in middle-aged men, either through a health promotion intervention alone or combined with an exercise intervention.

**Methods/design:**

During a two-year period we recruit 300 men aged from 35 to 45 years with elevated cardiovascular risk (> two traditional risk factors). The men are randomized into three arms: 1) a health promotion intervention alone, 2) both health promotion and exercise intervention, or 3) control with usual community care and delayed health promotion (these men receive the intervention after one year). The main outcome measures will be the existence of metabolic syndrome and physical activity frequency (times per week). The participants are assessed at baseline, and at 3, 6, and 12 months. The follow-up of the study will last 12 months.

**Discussion:**

This pragmatic trial in primary health care aimed to assess the effect of a health promotion programme with or without exercise intervention on cardiovascular risk and physical activity in middle-aged men. The results of this study may help to plan the primary care interventions to further reduce cardiovascular mortality.

The study was registered at the Controlled Trials ( http://www.controlled-trials.com). Trial number: ISRCTN80672011. The study received ethics approval from the Coordinating Ethics Committee at Helsinki University Hospital on 8 June 2009 (ref: 4/13/03/00/09).

## Background

Cardiovascular diseases continue to be among the most important causes of mortality, especially in men. In Finland, the North Karelia project is a success story of effective health promotion to reduce cardiovascular risk [1]. From a public health perspective, exercise is one the most effective means of reducing the risk of cardiovascular diseases. Exercise is a key modifiable factor either alone or in combination with other lifestyle changes and treatments to reduce hypertension and elevated cholesterol levels. The effectiveness of exercise has been shown in weight loss [2], particularly when combined with dietary changes. Exercise also decreases the incidence of type 2 diabetes mellitus in high risk groups (people with impaired glucose tolerance or the metabolic syndrome) [3]. There is also evidence that exercise improves the metabolic syndrome in general [4] and reduces HbA1c levels in patients with type 2 diabetes [5].

There is a lot of interest in cardiovascular risk reduction in primary care and many interventions are nurse-based [6,7]. Many communities in Finland have recently adopted health examinations by public health nurses for 40-year-old men to improve their lifestyle habits. Although the effectiveness of such interventions may now be questionable [8], at the planning stage of this project it was essential to include a nurse-based intervention. At the Kirkkonummi Health Center, we decided to combine a randomized controlled trial at the onset of such a health promotion project. The trial was approved by the municipality authorities.

A major challenge in health behavior changes is the maintenance of the adopted lifestyle [9]. An individual approach may not be sufficient for change. A group-based exercise intervention may motivate the participants to continue with their new exercise habits [10]. In addition to the health promotion intervention by a nurse, we included a group exercise intervention to increase the effectiveness of the intervention.

We aimed to develop an exercise intervention model for primary care that increases the physical activity of middle-aged men with increased cardiovascular risk. A randomized controlled trial was carried out to study the effects of this intervention. We also aimed to establish cooperation between a health center and other municipal activities, namely adult education and exercise services in health promotion of middle-aged men. The aim of this randomized controlled trial is to investigate the effects of this intervention on participants’ metabolic syndrome and physical activity.

## Objectives

The main aim of the study is to assess the effect of the interventions on physical activity and metabolic syndrome in middle-aged men, having at least two cardiovascular risk factors at the beginning of the trial.

The effect of the intervention on the individual risk factors of metabolic syndrome (overweight, waist circumference, blood cholesterol level, high-sensitivity C-reactive protein (hs-CRP)), on the physical activity factors (self-reported physical activity, and 2 km walking test), and body constitution as measured by InBody® are also examined.

## Methods/design

### Study design

**Figure 1:**
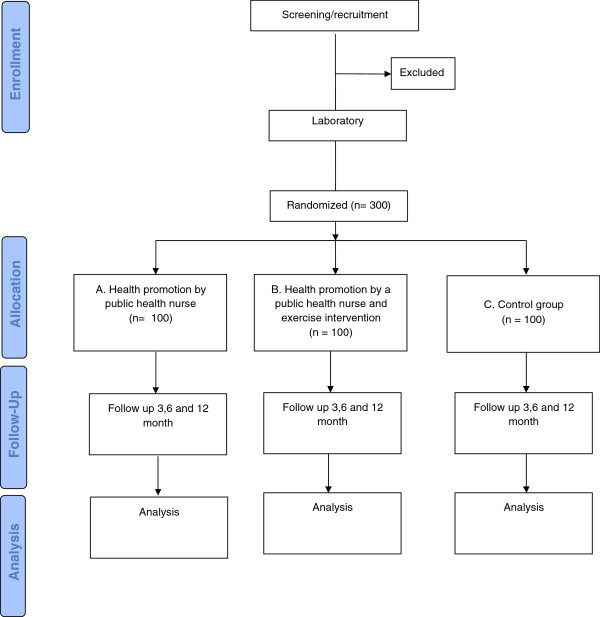
Study design and flow chart.

The study is a three-arm one-center randomized controlled trial (Figure 1). Two methods of health promotion are studied in middle-aged men with elevated cardiovascular risk. After screening and randomization, one study group receives a 1½-hour health promotion intervention by a public health nurse. The other intervention group receives, in addition to public health nurse intervention, a guided group exercise intervention of 12 weeks. The third group is randomized to serve as a control and men in this group may participate in the intervention after one year, should they wish to.

### Ethics approval

The study received ethics approval from the Coordinating Ethics Committee at Helsinki University Hospital on 8 June 2009 (ref: 4/13/03/00/09). All participants receive both oral and written participant information and are asked to give written informed consent.

### Recruitment and time schedule

We recruite men aged 35 to 45 years with at least two cardiovascular risk factors. They are recruited to the project in three ways: 1) by invitation letter to the 40-year-old age cohort, 2) by identifying high-risk group of men within health services (targeted opportunistic screening), and 3) by informing about the project in the media and on the Internet.

The study protocol was accepted in 2009 and recruitment of study subjects and randomization started in 2010.

#### Inclusion criteria

▪ Age 35–45 years

▪ At least two cardiovascular risk factors as follows:

- BMI 27–34 kg/m^2^

- Waist circumference > 94 cm

- Fasting glucose >6.1 mmol/l

- Total plasma cholesterol >4.0 mmol/l

- LDL-cholesterol > 3.0 mmol/l

- Triglycerides >2.0 mmol/l

- Systolic blood pressure > 140 mmHg

- Diastolic blood pressure > 90 mmHg

- Currently either :

• Smoking

• On cholesterol-lowering medication

• On blood pressure-lowering medication

#### Exclusion criteria

– BMI over 34 (or any other physical barrier preventing participation in the exercise intervention)

– Active exerciser (exercising at least 30 minutes 3 times a week or more)

– Having an immediate health problem requiring treatment or a severe risk factor; for example, recently diagnosed or uncontrolled type I diabetes, or a symptomatic coronary artery disease.

### Study interventions

In addition to study interventions, all groups receive, if necessary, standard treatment at Kirkkonummi Health Center, a municipal public primary care unit. If a participant shows any need of medical treatment, or his medications required medical attention, he is referred to a primary care physician.

### Health promotion intervention by nurse

Public health nurses use standard practices in their health promotion intervention. Before onset of the trial they receive education from the Finnish Heart Association. The nurses use the type 2 diabetes disease risk assessment form (Finnish Diabetes Association), and cardiovascular risk assessment (the Finnish Heart Association). The intervention includes assessment of individual risks, dietary habits, physical activity and a motivational talk about the habits. The intervention lasts up to 90 minutes and does not include follow-up.

If the men have elevated blood sugar, total plasma cholesterol over 7.0 mmol/l, uncontrolled hypertension or another reason for medical assessment, the nurse referres the patient to a physician for consideration of medication.

### Exercise intervention

In addition to the health promotion intervention, the other group is invited to a group exercise intervention. The size of the teaching groups is from 10 to 20 people. The exercise intervention is guided by a physical education counselor and consists of basic physical training. The exercise sessions each last 60 minutes. During the 12 weekly meetings several safe and moderately strenuous exercise activities are carried out, such as Nordic walking, fitness circle, volleyball, swimming, circuit training, gym, boxing, zumba, etc.

The exercise intervention aims at creating a comfortable atmosphere that encourages safe and regular physical training. Another aim is form groups of men that might continue exercising together.

### Control group

The participants of the control group receive both the health examination and the exercise intervention after the 12-month follow-up, if they so wish.

### Outcome measures

The outcomes are measured after 3, 6 and 12 months of the randomization. The blood tests are taken at months 0, 3 and 12. Other outcomes are measured by Internet surveys and, if the participant does not respond, by telephone surveys.

Primary outcome measures will be 1) metabolic syndrome defined by International Diabetes Federation/American Heart Association [11], thus fulfilling three or more of the following criteria:

- systolic blood pressure ≥ 130 or diastolic blood pressure ≥ 85 or specific medication;

- triglyserides ≥1.7 mmol/L or specific medication;

- HDL cholesterol < 1.0 mmol/L or specific medication

- fasting plasma glucose ≥ 5.6 mmol/L or specific medication;

- waist circumference >94 cm

2) and self-reported physical activity measured by how many times per week a participant performs vigorous exercise activity. Table 1 shows also the secondary outcome measures.

**Table 1:** Outcome measures

**Variable**	**T0**	**T1**	**T2**	**T3**
1. Primary outcome measures				
1.1 Metabolic syndrome1 (yes/no)	x	x	x	x
● Blood pressure, waist circumference,				
● Blood LDL- and HDL-cholesterol, triglycerides, blood glucose				
1.2 Physical activity	x	x	x	x
● Self-reported physical activity, exercise times, per week				
2. Other outcome measures				
● 2 km walking test (s)	x	x		x
● Self-reported general health (0–100)	x	x	x	x
● Self-reported overall stress (0–100)	x	x	x	x
● Utilisation and costs of medical services	x	x	x	x
● Individual cardiovascular risk factors: total cholesterol (mmol/L), LDL-cholesterol (mmol/L), HDL-cholesterol (mmol/L), smoking (yes/no), blood glucose (mmol/L), hs-CRP (mmol/L)	x	x	x	x
	x	x		x
● Body composition (InBody): muscle mass (kg), body fat mass (kg)	x	x		x

1 Defined by International Diabetes Federation/American Heart Association [11].

### Randomization

Once the men have consented to participate in the study and the baseline measurements are carried out, they are randomly allocated to one of the three study groups. A randomization list based on random numbers is made and transferred to sequentially numbered sealed envelopes.

### Sample size

Sample sizes were calculated by statistical power analysis. We hypothesised that the percentage of men continuing the increased exercise level after one year would be 30% in the control group and 50% in the exercise group. To detect this difference with α = .05 and β = .80, 91 men in each group are needed.

### Statistical analysis

A primary intention-to-treat analysis will be carried out. The main analyses involve standard two-sample comparisons (parametric or non-parametric depending on the distribution of the data) looking at effect sizes at 3 and 12 months.

### Qualitative analysis

In addition to quantitative methods, focus group discussions are conducted in order to study the experiences of the men in the intervention, the acceptability of the intervention and the barriers and facilitators for lifestyle change in men. The focus group discussions are held after the execution of the intervention. Using a qualitative approach we also study what the men’s experiences of the group exercise intervention are and whether they continue these activities after the trial. Also, the men’s preferred methods for joining projects aiming at lifestyle changes and other societal aspects are examined.

## Discussion

In this study, we assess the effects of primary care interventions aiming to reduce cardiovascular risk in early middle-aged men. Health promotion by public health nurses has become a popular method in cardiovascular prevention. Its effectiveness has, however, recently been questioned [4]. At the onset of this the trial, it was clear that it had to be included it in the intervention. We also wanted to study an exercise intervention, as many cardiovascular risk factors can be controlled through exercise [12,13].

We chose a group-based intervention because we considered the social support in the group to be an important motivational factor for the success of the intervention. Kirkkonummi is a suburban municipality, where most of the families move from other parts of Finland to work in the capital region. Many men do not have the social networks of their youth for maintaining their exercise activities. One possible result of this trial is that men continue to exercise in their intervention groups. We will follow-up for the potential societal effects of the trial using qualitative methods.

The strengths of this study are that it is a primary care pragmatic trial and that the results are readily generalizable to similar primary care settings. Also, the interventions included are feasible and non-expensive. If they prove effective, they can easily be transferred to other similar circumstances.

The study has some limitations as well. Blinding is not feasible and this study is performed as an open trial. Randomized controlled trials are not common in primary care health centers and there are threats to validity, e.g. how to guarantee similar processes by all study nurses and how to avoid other protocol violations. A special challenge is to keep the participants in the control group. As the participating men are not patients but working-aged and busy, their participation in the outcome measurements is a challenge.

The results of this study assess the effects of the interventions on physical activity and cardiovascular risk. We chose physical activity as a main outcome because from earlier literature we know that if men continue on their increased physical activity level, they will gain health benefits. The cardiovascular risk is a surrogate endpoint. It would, however, not be feasible to study morbidity because onset of cardiovascular risk will take years or decades in these men. In addition to these quantitative outcomes, assessing the participants’ views on barriers and facilitators to lifestyle changes after their experiences in the trial in the qualitative analysis will be of importance.

This study will provide information on the effects of an exercise intervention on cardiovascular risk and physical activity in middle-aged men. This pragmatic trial will shed light on the success of a physical activity intervention in real life, which is a recognized challenge in studies aiming at metabolic disease control [14]. The results of this study can be used in the planning of interventions aimed at reducing cardiovascular risk, diseases and mortality in primary care.

## Competing interests

Dr Pitkälä reports having professional cooperation including lecturing fees from pharmaceutical and other health care companies (including Lundbeck, MSD Finland, Novartis, Nestle), and having participated in clinical trials funded by pharmaceutical companies. Dr Liira has participated in Advisory Boards of Phizer and given lectures for Phizer and Abbott. Other authors declare no competing interests.

## Authors’ contributions

This study was carried out at the Kirkkonummi Health Center. Conception and design (HL, JL, TR-L, HT, KP), acquisition of data, or analysis and interpretation of data (SF, HL, JL, TR-L, HT, KP), drafting or critically revising the manuscript for important intellectual content (SF, HL, JL, TR-L, HT, KP); approval of the final manuscript (SF, HL, JL, TR-L, HT, KP KHP). KHP is the guarantor.

## Pre-publication history

The pre-publication history for this paper can be accessed here:

http://www.biomedcentral.com/1471-2458/13/125/prepub
